# A Course of Practical Chemistry

**Published:** 1870-02

**Authors:** 


A Course of Practical Chemistry Arranged for the use of Medical Students, by William Odling, M. B., F. R. S., etc. With illustrations. From the fourth London edition. Philadelphia: H. C. Lea. 1868. 1 vol. 8vo. cloth. Pp. 261.—An examination of this text-book convinces us that it will be a popular and useful one. It is not a deliberate treatise like that of Fownes, but a manual for the analysis and chemical examination of poisons, pathological matters, etc., which present themselves from time to time to the physician. Its style is clear and plain, the obscure nomenclature and symbolism which darken many chemical works very unnecessarily, being carefully avoided. We do not indeed know a more handy book of the kind for ordinary office use.

